# Let-7a-5p/SHIP-1 Axis Drives SARS-CoV-2 Spike-1-Induced Microglial Pyroptosis

**DOI:** 10.3390/ijms27177870

**Published:** 2026-09-03

**Authors:** Puja Pawar, Shraddha Ratnakar, Vandana Saxena

**Affiliations:** 1 ICMR National Institute of Translational Virology & AIDS Research, Pune 411026, India; pujappawar96@gmail.com (P.P.); shraddhartnkr@gmail.com (S.R.); 2 Department of Microbiology, Savitribai Phule Pune University, Pune 411007, India; 3 Faculty of Biological Sciences, Academy of Scientific and Innovative Research (AcSIR), Ghaziabad 201002, India

**Keywords:** SARS-CoV-2 spike, neuroinflammation, let-7a-5p/SHIP-1, pyroptosis, microglia

## Abstract

Although persistent neurological sequelae in long COVID are reportedly well associated with neuroinflammation, the underlying regulatory mechanisms remain poorly characterized. Previously, we identified dysregulated microRNA expression, including let-7a-5p, in severe acute respiratory syndrome coronavirus 2 (SARS-CoV-2) spike S1-stimulated human microglial cells by RNA sequencing; however, how let-7a-5p regulates the S1-mediated neuroinflammatory processes remains undetermined. In the present study, we examined the functional role of let-7a-5p in alleviating S1-induced microglial inflammation in the CHME3 cell line as well as in human monocyte-derived microglia (MDMi) using a loss- and gain-of-function approach. Functional inhibition of let-7a-5p resulted in mitigating S1-induced inflammatory cytokine release and markers of pyroptosis. Mechanistically, we established SHIP-1 as a direct target of let-7a-5p using luciferase reporter assay validation. Interestingly, we noted upregulated expression of the *TLR3* gene alongside *TLR2/4* in S1-stimulated microglia. Although we could not establish exactly how TLR3 is stimulated in S1-induced neuroinflammatory processes, using siRNA-mediated inhibition and a pharmacological inhibitor in both CHME3 cells and MDMi, our study certainly provides evidence of TLR3 involvement during S1-induced microglial inflammation, which needs further investigation. Together, these *in vitro* findings demonstrate that the let-7a-5p/SHIP-1 axis regulates S1-induced inflammatory cascades in CHME3 and MDMi cells, providing mechanistic insight into its role in SARS-CoV-2-associated neuroinflammation and warranting further validation in appropriate *in vivo/organoid* models.

## 1. Introduction

Severe Acute Respiratory Syndrome Coronavirus 2 (SARS-CoV-2), the causative agent of Coronavirus Disease 2019 (COVID-19), has been associated with a wide range of neurological manifestations, including cognitive dysfunction, fatigue, brain fog, sleep disturbances, anxiety, and depression [1,2]. These symptoms may persist beyond the acute phase of infection and are collectively referred to as long COVID; when predominantly involving the nervous system, these manifestations are commonly described as neuroCOVID. Emerging evidence from animal models and *in vitro* BBB systems suggests that the SARS-CoV-2 S1 protein may cross the blood–brain barrier and potentially contribute to neurological manifestations [3,4]. Spike protein has also been found in a limited number of postmortem human brain samples following infection with SARS-CoV-2, but the extent and duration of spike protein persistence in the human brain are unknown [5,6]. It is therefore important to understand the molecular mechanisms involved during SARS-CoV-2 S1-associated neuroinflammatory responses.

Among the glial cells, microglia, the resident innate immune cells of the central nervous system, respond to pathogen- and damage-associated molecular patterns through pattern recognition receptors (PRRs) [7]. SARS-CoV-2 infection has been shown to activate microglia, triggering robust inflammatory responses and subsequent cytopathic effects in both human microglial cells [8] and non-human primate models [9]. Pyroptosis is an inflammatory form of programmed cell death characterized by inflammasome activation, caspase-1 cleavage, gasdermin-mediated membrane pore formation, and the subsequent release of proinflammatory cytokines, particularly interleukin (IL)-1β and IL-18 [10]. Emerging evidence indicates that enhanced pyroptosis is associated with SARS-CoV-2 infection in the lungs of patients, suggesting that pyroptosis-associated inflammatory mechanisms may contribute to pulmonary hyperinflammation and tissue injury [11]. Studies suggested that in severe COVID-19, inflammasome activation and subsequent pyroptosis of alveolar macrophages and epithelial cells contributed to acute lung injury and acute respiratory distress syndrome (ARDS) [12]. In peripheral immune cells, SARS-CoV-2-induced inflammation has been linked to NLRP3 inflammasome activation, caspase-1 cleavage, and pyroptotic cell death [13,14]; however, the regulatory mechanisms governing S1-induced pyroptosis in microglial cells, particularly the role of microRNAs, remain poorly defined.

MicroRNAs (miRNA/miRs) are key post-transcriptional regulators that not only play critical roles in regulating inflammatory responses but also serve as potential biomarkers associated with severe COVID-19 disease [15,16]. However, unlike the peripheral inflammatory responses, much less is reported regarding their role(s) during the neuroCOVID condition and inflammatory responses. A previous study reported dysregulated expression of pro- and anti-neuroinflammatory miRNAs, including miR-148a and miR-590, in COVID-19 patients, potentially indicating a plausible role of microRNAs in mediating neuroinflammation [17,18]. Our previous in silico analysis identified microRNA let-7a-5p as significantly upregulated in S1-stimulated CHME3 microglial cells [19]; however, the functional significance of let-7a-5p and its downstream regulatory targets in S1-induced neuroinflammatory responses remains largely unknown.

Since SARS-CoV-2 spike S1-induced inflammatory responses in microglial cells are initiated through PRR-mediated signalling, identifying the upstream pathways that regulate S1-induced let-7a-5p expression and downstream inflammatory responses is important. Among the PRRs, toll-like receptors (TLRs) are predominantly involved in COVID-19 immunopathogenesis [20,21], where TLR4 and TLR2 are associated with neuroinflammatory processes in human and mice microglial cells [22,23], leading to long-term cognitive dysfunction. In contrast, while TLR3 signalling is well characterized in peripheral epithelial and immune cells during viral infections [24,25], its role in SARS-CoV-2 S1-mediated neuroinflammatory signalling in microglia remains largely unexplored. Given that dysregulated TLR3 activation has been linked to neurodegenerative disorders [26,27], its potential role in COVID-19-associated neuronal damage needs to be examined.

To address these gaps, using CHME3, a human microglial cell line, and monocyte-derived microglia-like cells (MDMi), this study investigated the involvement of let-7a-5p and TLR3-mediated signalling during S1-induced inflammatory responses. Furthermore, we sought to elucidate the downstream regulatory molecular axis orchestrating S1-driven pyroptosis.

## 2. Results

### 2.1. MicroRNA let-7a-5p Regulates the Neuroinflammatory Response Driven by SARS-CoV-2 S1

**Figure 1:**
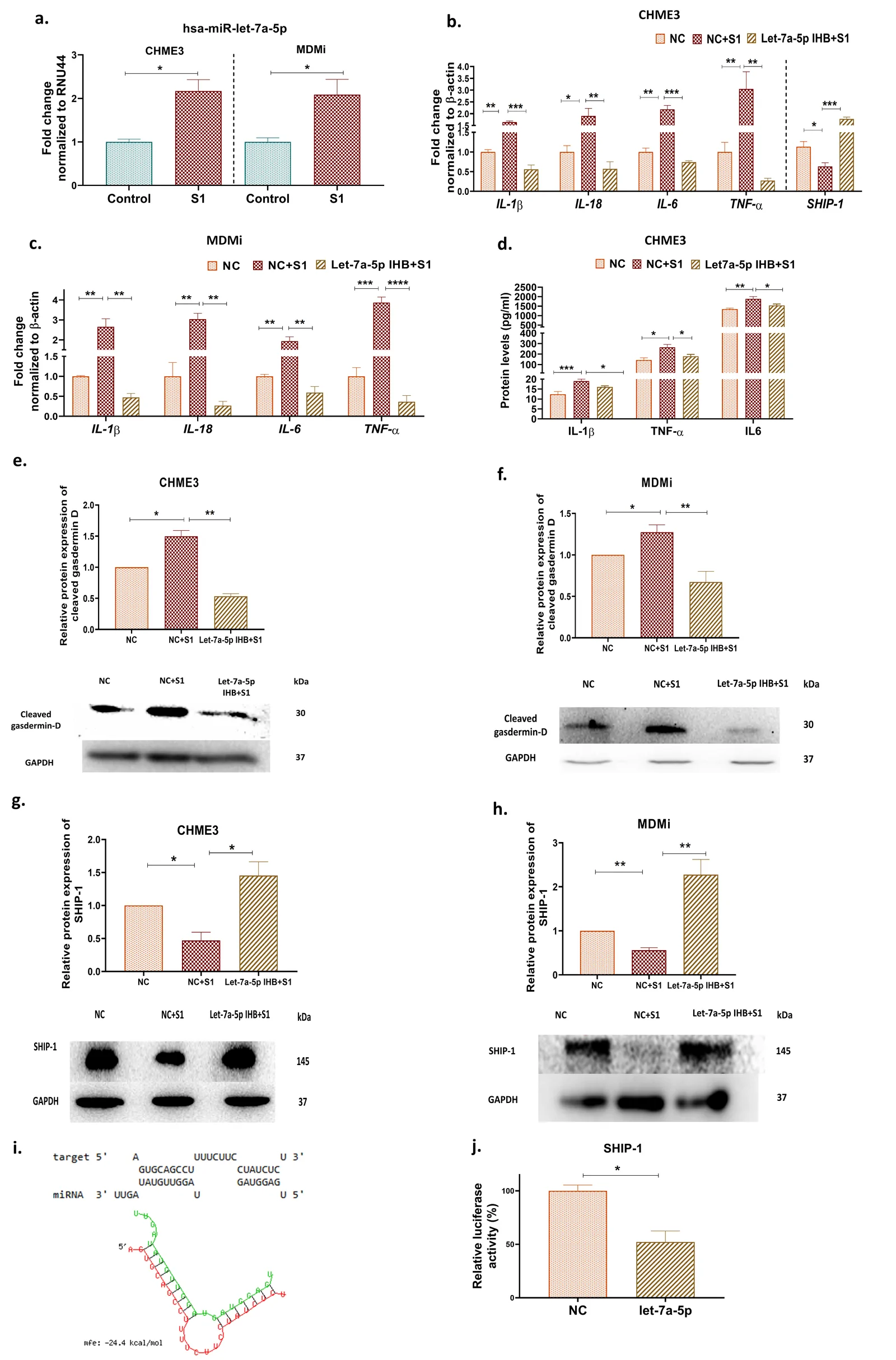
MicroRNA let-7a-5p regulates the neuroinflammatory response driven by SARS-CoV-2 S1. (**a**) RT-qPCR analysis of let-7a-5p expression in CHME3 and MDMi cells following SARS-CoV-2 spike S1 (150 ng/mL) stimulation. CHME3 and MDMi cells were transfected with the let-7a-5p inhibitor (IHB) or a scrambled negative control (NC) after S1 stimulation (NC + S1). (**b**,**c**) RT-qPCR analysis of *IL-1β, IL-18, IL-6, TNF-α, and SHIP-1* mRNA expression. (**d**) ELISA quantification of IL-1β and TNF-α in CHME3 culture supernatants. (**e**–**h**) Representative immunoblots and densitometric analysis of cleaved gasdermin D and SHIP-1 protein expression in CHME3 and MDMi cells; GAPDH served as the loading control. mRNA and protein expression in NC + S1 cells were normalized to NC, whereas let-7a-5p IHB+S1 cells were normalized to NC + S1. Statistical significance was determined using Student’s *t*-test or one-way ANOVA with Dunnett’s multiple-comparison test (* *p* < 0.05, ** *p* < 0.01, *** *p* < 0.001, **** *p* < 0.0001). (**i**) Predicted binding site of let-7a-5p within the SHIP-1 3′UTR, showing the miRNA–mRNA duplex and minimum free energy. (**j**) Relative luciferase activity of the pmirGLO–SHIP-1 reporter co-transfected with a let-7a-5p mimic or scrambled mimic (NC), expressed relative to NC.

In this study, we have used CHME3 cells as they are a well-established and reproducible human microglial model suitable for mechanistic studies, while MDMi cells were used as a human-derived model to provide supportive evidence with more physiological relevance and more closely mimic primary microglial phenotypes [28,29]. Consistent with our previous in silico analysis [19], S1 stimulation (150 ng/mL, 24 h) significantly increased let-7a-5p expression in both CHME3 cells (*p* = 0.01) and MDMi (*p* = 0.02) (Figure 1a). However, the detailed role of let-7a-5p in regulating the S1-induced inflammatory processes and pyroptosis is yet unidentified. Firstly, we confirmed knockdown of let-7a-5p expression in CHME3 cells post-transfection with the let-7a-5p inhibitor, compared with mock cells (transfected with a scrambled miRNA inhibitor) (Figure S2). We observed a significant decrease in let-7a-5p mRNA levels (*p* = 0.005, Figure S1) following let-7a-5p inhibition compared with the negative control (NC). Further, CHME3 cells and MDMi transfected with the let-7a-5p inhibitor had significantly decreased expression of inflammatory cytokines such as *IL-1β* (*p* = 0.0001, Figure 1b; *p* = 0.001, Figure 1c), *IL-18* (*p* = 0.008, Figure 1b; *p* = 0.002, Figure 1c), *IL-6* (*p* = 0.0006, Figure 1b; *p* = 0.002, Figure 1c), and *TNF-α* (*p* = 0.001, Figure 1b; *p* < 0.0001, Figure 1c) genes, while the expression of *SHIP-1*, a negative regulator of inflammatory processes, was found to be increased (*p* = 0.0002, Figure 1b) as compared to mock cells (transfected with a scrambled miRNA inhibitor) upon S1 stimulation. ELISA confirmed reduced secretion of IL-1β (*p* = 0.04), TNF-α (*p* = 0.04), and IL-6 (*p* = 0.04) at the protein level (Figure 1d). Furthermore, let-7a-5p inhibition significantly reduced the expression of S1-induced cleaved gasdermin D, a marker for pyroptosis (*p* = 0.002, Figure 1e,g; *p* = 0.006, Figure 1f). Similarly, SHIP-1 protein expression, which was inhibited post-S1 stimulation (*p* = 0.01, Figure 1g; *p* = 0.001, Figure 1h), was restored following let-7a-5p inhibition (*p* = 0.01, Figure 1g; *p* = 0.002, Figure 1h), unlike that of the unstimulated negative control (NC). Bioinformatic analysis identified a putative let-7a-5p binding site within the SHIP-1 3′UTR with a minimum free energy of −24.4 kcal/mol (Figure 1i). This interaction was further validated using a luciferase reporter assay, where the let-7a-5p mimic significantly reduced SHIP-1 3′UTR reporter activity compared with the control mimic (*p* = 0.02, Figure 1j) in HEK-293 cells, suggesting that SHIP-1 is a direct target gene of let-7a-5p. These data suggest that upregulation of let-7a-5p promotes S1-induced inflammatory responses and pyroptosis-associated signalling in these *in vitro* microglial models.

### 2.2. TLR3 Inhibition Prevents SARS-CoV-2 S1-Induced Inflammatory Response and Inflammasome-Mediated Cell Death/Pyroptosis in Human Microglial Cells

**Figure 2:**
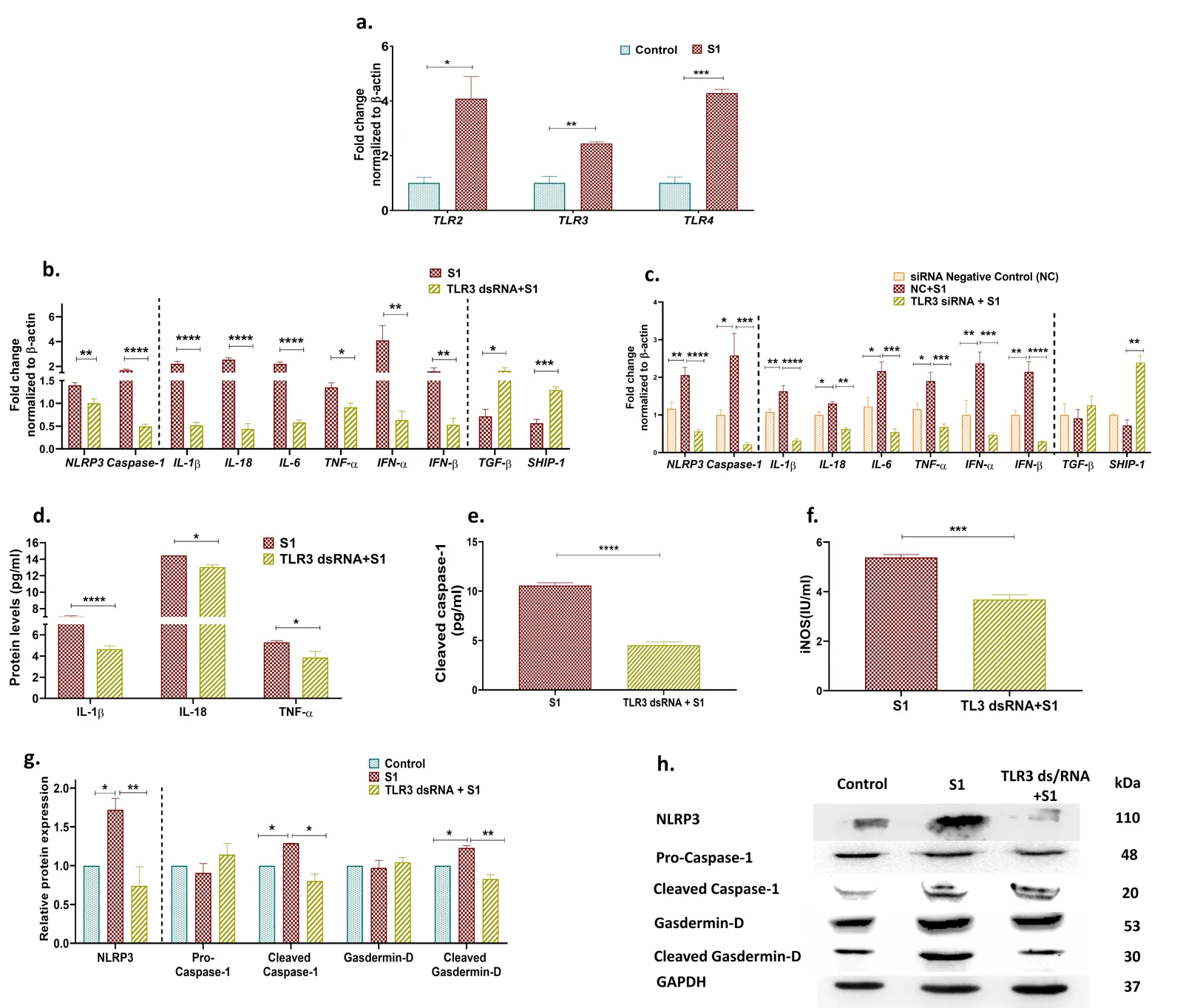
TLR3 inhibition prevents SARS-CoV-2 S1-induced inflammatory response and inflammasome-mediated cell death/pyroptosis in human microglial cells. (**a**) Relative mRNA expression of *TLR2, TLR3,* and *TLR4* following S1 stimulation (150 ng/mL, 24 h). (**b**,**c**) mRNA expression of *NLRP3, caspase-1, IL-1β, IL-18, IL-6, TNF-α, IFN-α, IFN-β, TGF-β,* and *SHIP-1* following pharmacological inhibition or siRNA-mediated knockdown of TLR3 prior to S1 stimulation. (**d**–**f**) Protein levels of IL-1β, IL-18, TNF-α, cleaved caspase-1, and iNOS following TLR3 inhibition. (**g**,**h**) Representative immunoblots and densitometric analysis of NLRP3, pro-caspase-1, cleaved caspase-1, gasdermin D, and cleaved gasdermin D. Gene expression was normalized to β-actin and protein expression to GAPDH, and fold changes were calculated relative to the indicated control groups. Data are presented as mean ± SEM. Statistical significance was determined using an unpaired Student’s *t*-test or one-way ANOVA with Dunnett’s post hoc test. * indicates a *p* value < 0.05, ** indicates a *p* value < 0.01, *** indicates a *p* value < 0.001, **** indicates a *p* value < 0.0001.

S1 stimulation in CHME3 and MDMi induced a significant increase in the expressions of *IL-1β* (*p* = 0.002, *p* < 0.0001), *IL-18* (*p* < 0.0001, *p* = 0.003), *IL-6* (*p* = 0.0003, *p* = 0.02), *TNF-α* (*p* = 0.03, *p* = 0.02), *IFN-α* (*p* = 0.05, *p* = 0.002) and *IFN-β* (*p* = 0.04, *p* = 0.012) compared with the non-stimulated cells (Figure S3a,f). The protein expression profiles of the inflammatory cytokines IL-1β (*p* = 0.03), TNF-α (*p* = 0.04), and IL-18 (*p* = 0.02), along with cleaved caspase-1 (*p* = 0.0004) and iNOS expression (*p* = 0.0005), were increased in S1-stimulated CHME3 cells compared with non-stimulated cells (Figure S3c,e). Using a multiplex protein assay, increased expression levels of IL-1β (*p* = 0.01), IL-6 (*p* = 0.008), TNF-α (*p* = 0.02) and IFN-α (*p* = 0.05) were noted in the S1-stimulated MDMi compared with the non-stimulated cells (Figure S3g). We also noted increased expression of the chemokines IL8 (*p* = 0.002), GM-CSF (*p* = 0.01) and CCL2 (*p* = 0.03) in the S1-stimulated MDMi compared with that of control cells (Figure S3h). Interestingly, the expression profiles of anti-inflammatory genes *TGF-β* and *SHIP-1* were significantly decreased in both S1-stimulated CHME3 cells (*p* = 0.05 and *p* = 0.003, respectively; Figure S3a) and MDMi (*p* = 0.03 and *p* = 0.03, respectively; Figure S3f). In addition, a significant decrease in the protein level of TGF-β in both S1-stimulated CHME3 cells (*p* = 0.04; Figure S3d) and MDMi (*p* = 0.02; Figure S3h) was observed. As S1 stimulation induced robust inflammatory responses in both CHME3 cells and MDMi, we next investigated the upstream PRRs involved. We first examined the expression of TLRs following S1 stimulation. In CHME3 cells, *TLR2* (*p* = 0.02), *TLR3* (*p* = 0.005), and *TLR4* (*p* = 0.0002) genes were observed post-S1 stimulation (Figure 2a), while no change was observed in *TLR-7, -8, or -9* genes. Compared with other TLRs, the substantial roles of TLR2- and TLR4-mediated processes in proinflammatory cytokine release have been documented during SARS-CoV-2-induced inflammation associated with respiratory and neurological complications [30,31,32]. However, the precise function of TLR3 in SARS-CoV-2 S1-mediated neuroinflammatory response and its association with pyroptosis is hitherto undefined, which we examined using (i) a pharmacological TLR3/dsRNA complex inhibitor and (ii) siRNA-mediated inhibition of TLR3, before S1 stimulation in CHME3 cells as well as in MDMi. In CHME3 cells, significant decreases in the gene expression of *NLRP3* (*p* = 0.009, *p* < 0.0001), *caspase-1* (*p* < 0.0001, *p* = 0.0008) and inflammatory cytokines *IL-1β* (*p* < 0.0001, *p* < 0.0001), *IL-18* (*p* < 0.0001, *p* = 0.002), *IL-6* (*p* < 0.0001, *p =* 0.0002), *TNF-α* (*p* = 0.01, *p* = 0.0005), *IFN-α* (*p* = 0.007, *p* = 0.0007), and *IFN-β* (*p* = 0.008, *p* < 0.0001) and significant increases in the expression of anti-inflammatory genes such as *TGF-β* (*p* = 0.02) and *SHIP-1* (*p* = 0.0003, *p* = 0.001) were noted after treatment with both the TLR3/dsRNA complex inhibitor (Figure 2b) and TLR3 siRNA (Figure 2c) compared to the mock cells (S1 stimulation), respectively, whereas no significant difference was observed in the gene expression of *TGF-β* after using TLR3 siRNA (Figure 2c). Interestingly, we noted significant decreases in the protein levels (Figure 2d) of IL1β (*p* < 0.0001), IL-18 (*p* = 0.04), TNF-α (*p* = 0.03), cleaved caspase-1 (*p* < 0.0001, Figure 2e), and iNOS (*p* = 0.0005, Figure 2f) compared to mock cells. To confirm that S1-induced inflammatory responses were associated with inflammasome activation, we first demonstrated that post-S1 stimulation, *NLRP3* (*p* = 0.01) and *caspase-1* (*p* = 0.002) mRNA expression was significantly increased in CHME3 cells as compared to the control (Figure S4a). Further protein data analysis using Western blotting (Figure S4e,f) showed that S1 stimulation increased expression of NLRP3 (*p* = 0.0002), cleaved caspase-1 (*p* = 0.01), and cleaved gasdermin D (*p* = 0.02), while pro-caspase-1 and gasdermin D expression remained unchanged. To further confirm the role of NLRP3, cells were first treated with MCC950 (inhibitor of NLRP3 activation), followed by stimulation with S1 protein. Blocking with MCC950 significantly decreased the expression of NLRP3 (*p* < 0.0001), cleaved caspase-1 (*p* = 0.002), and cleaved gasdermin D (*p* = 0.003) in the CHME3 cells post-S1 stimulation (Figure S4e,f). We next inhibited caspase-1 by treatment with a pan-caspase inhibitor (Z-VAD-FMK). Indeed, we observed a significant reduction in the cleaved caspase-1 (*p* = 0.005) and cleaved gasdermin D (*p* = 0.008) expression post-S1 stimulation in the cells treated with Z-VAD-FMK compared with the S1-stimulated cells only (mock) (Figure S4e,f). In addition, treatment with pharmacological inhibitors of NLRP3 (MCC950) and caspases (Z-VAD-FMK) decreased the protein levels of proinflammatory cytokines (Figure S4b), i.e., IL-1β (*p* = 0.0008, *p* = 0.0003) and IL-18 (*p* = 0.02), respectively, while no change was observed in IL-18 levels post-Z-VAD-FMK treatment. Interestingly, significant decreases in cleaved caspase-1 (*p* = 0.0034, *p* = 0.0007; Figure S4c) and iNOS were noted upon treatment with MCC950 (*p* = 0.006) and Z-VAD-FMK (*p* = 0.0002) post-S1 stimulation (Figure S4d). Similarly, TLR3 inhibition revealed a significant decrease in the protein levels of NLRP3 (*p* = 0.009), cleaved caspase-1 (*p* = 0.01), and cleaved gasdermin D (*p* = 0.002) compared to mock cells according to densitometry analysis (Figure 2g,h), whereas no change in the levels of pro-caspase-1 and gasdermin D was observed (Figure 2g,h).

**Figure 3:**
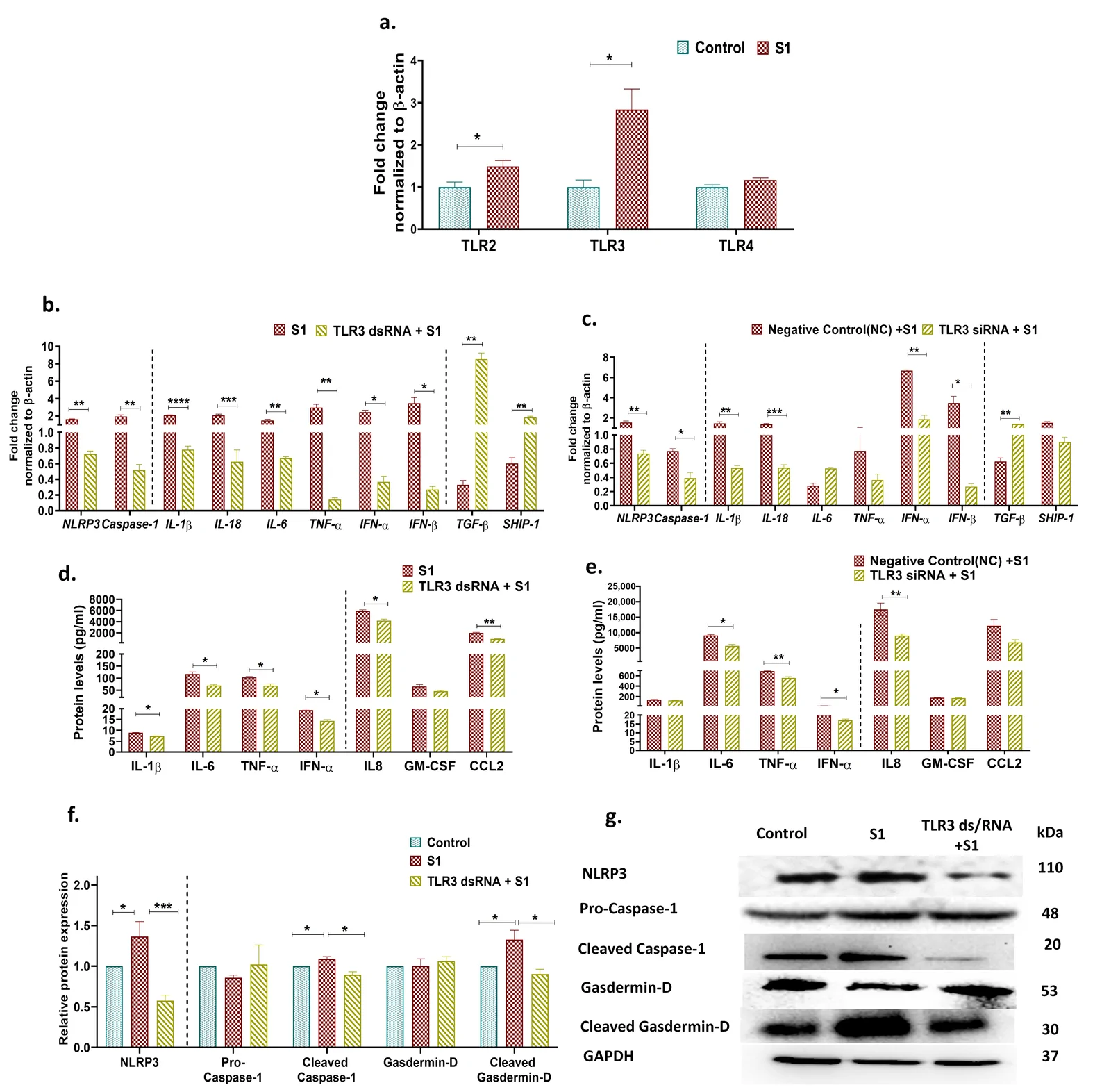
TLR3 inhibition prevents the SARS-CoV-2 S1-induced inflammatory response and inflammasome-mediated cell death/pyroptosis in human microglial cells. (**a**) Relative mRNA expression of *TLR2*, *TLR3,* and *TLR4* following S1 stimulation (150 ng/mL, 24 h). (**b**,**c**) mRNA expression of *NLRP3, caspase-1, IL-1β, IL-18, IL-6, TNF-α, IFN-α, IFN-β, TGF-β*, and *SHIP-1* following pharmacological inhibition or siRNA-mediated knockdown of TLR3 before S1 stimulation. (**d**,**e**) Protein levels of IL-1β, IL-6, TNF-α, IFN-α, IL-8, GM-CSF, and CCL2 were measured by a Luminex assay. (**f**,**g**) Representative immunoblots and densitometric analysis of NLRP3, pro-caspase-1, cleaved caspase-1, gasdermin D, and cleaved gasdermin D. Gene expression was normalized to β-actin and protein expression to GAPDH, and fold changes were calculated relative to the indicated control groups. Data are presented as mean ± SEM. Statistical significance was determined using an unpaired Student’s *t*-test or one-way ANOVA with Dunnett’s post hoc test. * indicates a *p* value < 0.05, ** indicates a *p* value < 0.01, *** indicates a *p* value < 0.001, **** indicates a *p* value < 0.0001.

To corroborate the foregoing results, we next evaluated the expression of TLRs in S1-stimulated MDMi and noted a significant increase in the expression of *TLR2* (*p* = 0.04) and *TLR3* (*p* = 0.02) genes (Figure 3a), while no change in the gene expression of *TLR-4, -7, -8, or -9* was detected post-24 h. Both pharmacological inhibition and siRNA-mediated knockdown of TLR3 attenuated S1-induced expression of inflammasome-associated genes including *NLRP3* (*p* = 0.006, *p* = 0.002) and *caspase-1* (*p* = 0.002, *p* = 0.03) along with inflammatory cytokines *IL-1β* (*p* < 0.0001, *p* = 0.001), *IL-18* (*p* = 0.0007, *p* = 0.0001), *IL-6* (*p* = 0.004, Figure 3b), *TNF-α* (*p* = 0.002, Figure 3b), IFN-α (*p* = 0.01, *p* = 0.002), and *IFN-β* (*p* = 0.04, *p* = 0.04), and significant increases in the expression of anti-inflammatory genes such as *TGF-β* (*p* = 0.007, *p* = 0.005) and *SHIP-1* (*p* = 0.001; Figure 3b) were observed as compared to mock cells, whereas no significant difference in the gene expression of *IL-6*, *TNF-α*, or *SHIP-1* was observed after TLR3 knockdown with siRNA compared to mock cells (Figure 3c). Interestingly, we noted a significant decrease in the protein levels of IL-1β (*p* = 0.0194, Figure 3d), IL-6 (*p* = 0.04, Figure 3d; *p* = 0.02, Figure 3e), TNF-α (*p* = 0.05, Figure 3d; *p* = 0.006, Figure 3e), IFN-α (*p* = 0.03, Figure 3d; *p* = 0.01, Figure 3e), IL-8 (*p* = 0.02, Figure 3d; *p* = 0.006, Figure 3e), and CCL2 (*p* = 0.001, Figure 3d) compared to mock cells. However, upon inhibition using TLR3 siRNA, the protein levels of IL-1β, GM-CSF, and CCL2 (Figure 3e) showed no significant difference (Figure 3d–g). Collectively, these findings suggest that TLR3 signalling contributes to S1-induced inflammasome activation and pyroptosis-associated inflammatory responses in *in vitro* microglial models.

## 3. Discussion

Persistent neurological symptoms following COVID-19 have been linked to sustained neuroinflammatory processes that may occur independently of active viral replication, particularly given reports that the SARS-CoV-2 spike (S1) protein persists in brain tissues long after viral clearance [6]. Studies have shown that S1 induced microglial activation with downstream inflammatory and oxidative-stress responses [33,34], providing a rationale for investigating the underlying molecular mechanisms in human microglial models. Using CHME3 cells and MDMi, the present *in vitro* study examined the role of microRNA let-7a-5p in regulating S1-induced neuroinflammatory responses. Our findings provide evidence that let-7a-5p, via its validated target SHIP-1, contributes to S1-induced cytokine release and pyroptotic markers in these models.

Consistent with our prior in silico observation [19], S1 stimulation upregulated let-7a-5p in both CHME3 and MDMi, and loss-of-function experiments supported a proinflammatory role: let-7a-5p inhibition reduced S1-induced IL-1β, IL-18, IL-6, and TNF-α expression, decreased cleaved gasdermin D, and restored SHIP-1 protein expression. Luciferase reporter assays confirmed direct targeting of the SHIP-1 3′UTR by let-7a-5p. These observations are in line with reports of let-7a/b driving proinflammatory cytokines via TLR7 in microglia during Japanese encephalitis virus infection [35], in chondrocytes upon LPS stimulation [36], and also in systemic lupus erythematosus (SLE) [37]. Furthermore, SHIP-1 is a well-established negative regulator of inflammatory signalling [38,39] and has been identified as a target of several microRNAs, including miR-155 and miR-330 [40,41]. Notably, let-7a-5p expression appears context-dependent in COVID-19, being elevated in plasma [42] but reduced in saliva [43], underscoring the importance of cellular context. To the best of our knowledge, this is the first study to provide functional evidence for the let-7a/SHIP-1 axis in S1-stimulated human microglial models; however, confirmatory work in an *in vivo* infection model is required to determine its relevance to COVID-19-associated neuroinflammation. Further, in the present study, a dual-luciferase reporter assay was performed in HEK293 cells, a commonly used model for miRNA–3′UTR interaction studies due to high transfection efficiency and suitability for quantitative reporter assays [44]. Additionally, transfection in microglial cells can be relatively difficult and may require specialized approaches [45], posing a limitation to our findings. Future validation studies using optimized methods in human microglial or monocyte-derived microglial models will further validate this regulatory axis in a physiologically relevant context.Microglia are the resident macrophages of the brain, where pathogen recognition receptors, particularly TLR-mediated signalling, are critically involved in driving downstream inflammatory events. Among the PRRs examined, *TLR2* and *TLR3* were upregulated in both models, while *TLR4* was induced only in CHME3. TLR2- and TLR4-mediated signalling in S1-induced inflammation is well documented [22,32,46], whereas the role of TLR3 in S1-stimulated microglia has been less well defined. Both pharmacological inhibition and siRNA-mediated knockdown of TLR3 reduced S1-induced NLRP3, cleaved caspase-1, cleaved gasdermin D, and a panel of inflammatory cytokines and chemokines, consistent with the reported involvement of TLR3–NLRP3/NF-κB/IRF3 signalling in viral infections such as Respiratory Syncytial Virus and Enterovirus [47,48] and TLR3 activation in neuroinflammatory disorders [27,49]. The role of NLRP3 and caspase-1 in mediating pyroptosis during S1-induced neuroinflammation was further confirmed by using their pharmacological inhibitors, i.e., MCC950 and Z-VAD-FMK, respectively. In response to S1 stimulation, a significant decrease in cleaved gasdermin D, along with inflammatory cytokines such as IL-1β, IL-18, and iNOS, was noted when NLRP3 and caspase-1 were inhibited, which is consistent with prior reports of S1-mediated NLRP3 activation in microglia [34,50] and with NLRP3-mediated pyroptosis in monocytes from COVID-19 patients [13,51]. Our findings extend these observations to *in vitro* human microglial models but do not exclude the contribution of other cell death pathways, which warrant further investigation. Our results support the involvement of pyroptosis-associated signalling in S1-induced neuroinflammation; however, direct evidence of terminal pyroptotic cell death, such as membrane permeabilization or characteristic morphological changes, was not assessed in the present study and needs further investigation. Because S1 is not a canonical TLR3 ligand, the mechanism of TLR3 engagement remains speculative; indirect routes involving endogenous RNA release, altered RNA metabolism, or stress responses have been proposed [52,53], but they require further investigation. Furthermore, increasing evidence suggests that miRNAs are not only post-transcriptional regulators of gene expression, but also important regulators of TLR signalling through interactions with TLR-associated signalling molecules and downstream pathways such as NF-κB and JAK-STAT signalling [54,55]. Interestingly, the let-7 family has a dual function by regulating the expression of inflammatory genes and acting as endogenous ligands for TLRs, modulating innate immune responses. The let-7 family has been demonstrated to induce TLR7-dependent signalling, resulting in microglial activation and neuroinflammatory responses [56]. In line with these, our findings also suggest the involvement of the let-7a-5p/SHIP-1 axis in the regulation of TLR-mediated inflammatory responses and pyroptosis in microglial cells. However, further studies are warranted to determine whether let-7a-5p directly interacts with TLR-dependent signalling pathways during SARS-CoV-2-induced neuroinflammation.

Multiple factors are involved in the pathophysiology of COVID-19-associated neuroinflammation, including viral replication, several viral proteins (S1, N, E, M, and accessory proteins), systemic cytokine responses, hypoxia, and endothelial dysfunction, etc. [57,58]. The present study specifically identified spike-1-driven alterations in the molecular mechanisms resulting in increased pyroptosis, which are likely to contribute towards the overall neuroinflammatory cascade, but they do not comprehensively explain the multifactorial neuropathology observed in COVID-19-associated neuroinflammation *in vivo*. Validation of these pathways in more physiologically complex systems such as whole-virus infection models or ACE2-humanized animal models [59] will be essential to establish their relative *in vivo* significance.

**Figure 4:**
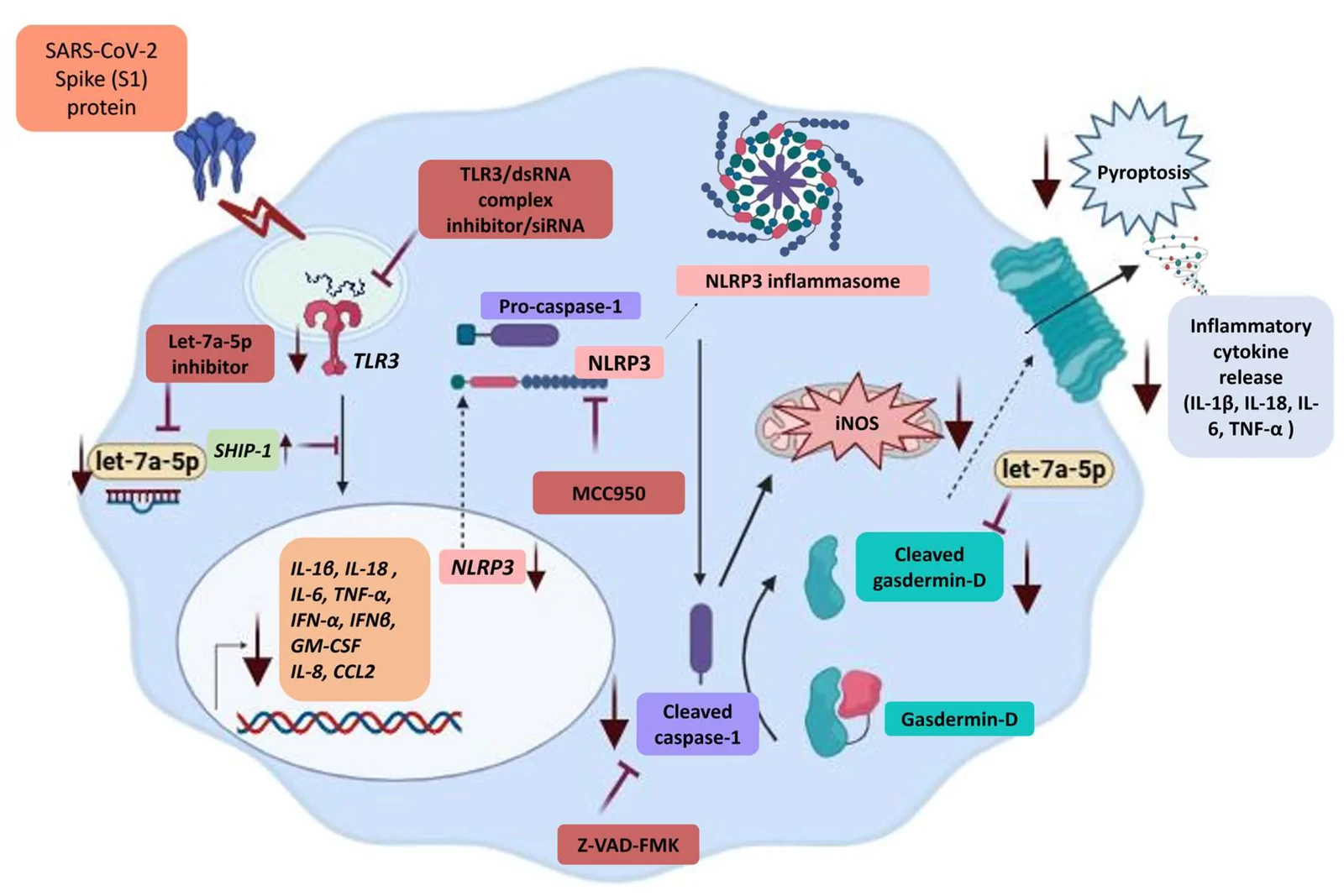
A graphical representation showing that SARS-CoV-2 spike (S1) induced a neuroinflammatory response and pyroptosis, mediated through TLR3/NLRP3 activation and the regulatory role of the let-7a-5p/SHIP-1 axis.

Overall, the present study provides evidence that the let-7a-5p/SHIP-1 axis modulates SARS-CoV-2 S1-induced inflammatory responses and pyroptosis in CHME3 and human monocyte-derived microglial cell models, with TLR-3 facilitating inflammasome-mediated pyroptosis and oxidative stress in human microglial cells (Figure 4). The data support a model in which S1 stimulation upregulates let-7a-5p, which in turn suppresses the negative regulator SHIP-1, thereby facilitating pyroptotic signalling. Although SHIP-1 was identified as a direct and functionally relevant target of let-7a-5p in our study, previous studies have indicated that let-7a-5p can regulate multiple inflammation-associated targets including MYD88, IL-6, and MAP3K in different disease conditions, suggesting that additional downstream targets may contribute to its inflammatory effects [60,61,62]. Therefore, the let-7a-5p/SHIP-1 axis should be considered one of the mechanisms contributing to the observed inflammatory response rather than the only regulatory pathway. While a SHIP-1 rescue experiment was beyond the scope of the present study, future rescue experiments in microglial cells will be important to further establish the specific role of SHIP-1 in the let-7a-5p-mediated inflammatory response.

Importantly, the overall inflammatory response showed similarities between CHME3 and MDMi cells; however, TLR4 upregulation was observed in CHME3 cells but not in MDMi cells, reflecting the biological heterogeneity between immortalized and primary-derived microglial models. In addition, some cytokine (IFN-α and GM-CSF) and chemokine (CCL2 and IL-8) profiling was performed in MDMi cells and therefore provides complementary evidence from the primary-derived microglial model rather than a direct comparison between the two models. These findings emphasize the importance of considering model-specific characteristics when interpreting microglial responses to SARS-CoV-2 S1. Within the limitations of this *in vitro* approach, these findings contribute to the understanding of potential regulatory mechanisms in S1-stimulated microglial models and suggest that the let-7a-5p/SHIP-1 axis and TLR3 may represent candidates for further investigation in *in vivo* models of SARS-CoV-2-associated neuroinflammation.

## 4. Materials and Methods

### 4.1. Cell Culture and Generation of Human Monocyte-Derived Microglia (MDMi)

CHME3 human microglial cells were maintained in DMEM supplemented with 10% heat-inactivated FBS and penicillin–streptomycin at 37 °C in 5% CO_2_. For the differentiation of human MDMi, peripheral blood mononuclear cells (PBMCs) isolated from the buffy coats of healthy donors (*n* = 5–7) were used. Buffy coats were obtained from a blood bank, which collects blood after obtaining informed consent from healthy volunteers. Using the plastic adherence method as described in previous studies [63,64], MDMi were differentiated from PBMCs. Briefly, PBMCs were isolated from buffy coats through Ficoll-Hypaque density-gradient centrifugation and cultured overnight in RPMI-1640 including 10% FBS and penicillin–streptomycin (10,000 U/mL) at 37 °C with 5% CO_2_. The following day, non-adherent cells were removed, and the adherent monocytes were differentiated into microglia-like cells by culturing them for 10 days in serum-free RPMI-1640 medium supplemented with the following human recombinant cytokines: M-CSF (10 ng/mL), GM-CSF (10 ng/mL), β-NGF (20 ng/mL) and CCL2 (100 ng/mL; Peprotech, Cranbury, NJ, USA). The differentiation medium was refreshed every two days with fresh serum-free RPMI-1640 containing the corresponding recombinant cytokines at the indicated concentrations. The culture media and all supplements necessary for the cell culture were sourced from Gibco, Thermo Fisher Scientific, Waltham, MA, USA. Differentiation was confirmed by flow cytometric analysis of the microglial marker P2RY12 (Biolegend, San Diego, CA, USA) [65] (Figure S1).

### 4.2. CHME3 and MDMi Treatment with Inhibitors, TLR3 siRNA and hsa-let-7a-5p Inhibitor Transfection

CHME3 cells and MDMi were stimulated with recombinant SARS-CoV-2 spike S1 protein (150 ng/mL; Abcam, Cambridge, UK) for 24 h. The concentration of the S1 protein (150 ng/mL) and the time of stimulation (24 h) were selected on the basis of our previous study [19] demonstrating robust and reproducible cellular responses under these experimental conditions. These established parameters were therefore used in the current study to promote consistency and reproducibility between experiments.

For inhibition studies, cells were pre-treated with an NLRP3 inhibitor—MCC950 sodium (20 μM; MedChem Express, Monmouth Junction, NJ, USA)—a pan-caspase inhibitor—Z-VAD-FMK (20 μM; R&D Systems, Minneapolis, MN, USA)—or the TLR3/dsRNA complex inhibitor (40 μM; Sigma-Aldrich, St. Louis, MO, USA) for 60 min before S1 stimulation. For siRNA-mediated knockdown, CHME3 cells and MDMi at 70–80% confluence were transfected with TLR3-targeting siRNA using HiPerFect (Qiagen, Hilden, Germany) or Silencer™ negative control siRNA (150 nM) (Thermo Fisher Scientific, MA, USA) following the manufacturer’s protocol. For let-7a-5p inhibition, CHME3 cells were transfected with 150 nM hsa-let-7a-5p inhibitor (Thermo Fisher Scientific, MA, USA) or 150 nM scrambled miR inhibitor (negative control; Dharmacon, Lafayette, CO, USA) using HiPerFect, followed by S1 (150 ng/mL) stimulation for 24 h. Following successful transfection, cells were stimulated with SARS-CoV-2 S1 protein (150 ng/mL) for 24 h. cDNA synthesis was performed using a Reverse Transcription Kit from TaqMan™. MicroRNA hsa-let-7a-5p inhibition was determined by real-time (RT) PCR using Universal Master Mix-II and microRNA assays (TaqMan™) for hsa-let-7a-5p, and RNU-44 (Applied Biosystems™, Foster City, CA, USA). Following treatment, cells and culture supernatants were collected for gene expression, protein, and cytokine analyses.

### 4.3. RNA Extraction, cDNA Synthesis and Quantitative Real-Time PCR (RT-PCR)

Total RNA was extracted using TRIzol reagent (Invitrogen, Thermo Fisher Scientific, MA, USA) according to the manufacturer’s instructions. RNA quantity and purity were assessed using a NanoDrop 2000 spectrophotometer (Thermo Fisher Scientific, MA, USA). cDNA was synthesized using the PrimeScript Reverse Transcription Kit (Takara Bio Inc., Kusatsu, Japan). Quantitative real-time PCR (qRT-PCR) was performed using PowerUp™ SYBR™ Green Master Mix on a QuantStudio™ 6 Pro Real-Time PCR System (Applied Biosystems, MA, USA). Expression levels of IL-1β, IL-18, TNF-α, IL-6, and SHIP-1 were analyzed using gene-specific primers custom-designed and obtained from Integrated DNA Technologies (IDT), Coralville, IA, USA (Table S1). Relative gene expression was normalized to β-actin and fold change was calculated using the 2^−ΔΔCt^ method.

### 4.4. Multiplex Cytokine Analysis and Enzyme-Linked Immunosorbent Assay (ELISA)

Cytokine secretion in MDMi culture supernatants was quantified using the ProcartaPlex™ Human Inflammation Panel 20-Plex (Invitrogen, Thermo Fisher Scientific, MA, USA) and analyzed on a Bio-Plex 200 system (Bio-Rad, Hercules, CA, USA) according to the manufacturer’s instructions. Cytokine concentrations were calculated from standard curves and expressed as pg/mL.

For CHME3 cells, concentrations of IL-1β, IL-18, TNF-α, IL-6, and inducible nitric oxide synthase (iNOS) in culture supernatants were determined using commercially available ELISA kits (R&D Systems and Cusabio, Houston, TX, USA) following the manufacturers’ protocols. Absorbance was measured at 450 nm using a TECAN Sunrise microplate reader (Tecan, Männedorf, Switzerland), and concentrations were calculated from standard curves. The final concentrations were expressed as pg/ml for cytokines and IU/ml for iNOS.

### 4.5. Western Blot Analysis for Protein Expression

Total cellular proteins were extracted from CHME3 and MDMi cells using RIPA buffer supplemented with protease inhibitors (Thermo Fisher Scientific, MA, USA). Protein concentrations were determined using the Bradford protein reagent (Thermo Fisher Scientific, MA, USA). Total protein (100 μg) was separated by SDS-PAGE, transferred onto PVDF membranes, and probed with antibodies against NLRP3, caspase-1, cleaved caspase-1, gasdermin D, cleaved gasdermin D, SHIP-1, and GAPDH (Cell Signaling Technology, Danvers, MA, USA). All the bands were visualized using enhanced chemiluminescence on a ChemiDoc Imaging System (Bio-Rad, CA, USA). Band intensities were quantified using ImageJ software (V1.54k) and normalized to GAPDH and expressed as fold change over the respective control group.

### 4.6. miRNA-mRNA Target Prediction and Validation by Luciferase Reporter Assay

Potential binding of hsa-let-7a-5p to the SHIP-1 3′UTR was predicted using the RNAhybrid server (https://bibiserv.cebitec.uni-bielefeld.de/rnahybrid (accessed on 2 April 2025)) [66,67] based on sequences obtained from the UCSC Genome Browser (GRCh38 build; https://genome.ucsc.edu/ (accessed on 2 April 2025)) [68] and from miRBase (https://www.mirbase.org/ (accessed on 2 April 2025)) [69]. The SHIP-1 3’UTR sequence was custom-synthesized in the luciferase reporter vector pmiRGLO. HEK293 cells were co-transfected with reporter plasmid (350 ng) and a let-7a-5p mimic (Thermo Fisher Scientific, MA, USA) or scrambled oligonucleotide mimic (negative control) (Dharmacon, CO, USA) using jetPRIME (Polyplus, Berkeley, CA, USA) transfection reagent. After 48 h, luciferase activity was measured using the Bright-Glo™ Luciferase Assay System (Promega, Fitchburg, WI, USA).

### 4.7. Statistical Analysis

Data are presented as mean ± SEM from 3–5 independent experiments. Statistical analyses were performed using GraphPad Prism v8.4.2 (GraphPad Software, San Diego, CA, USA). Comparisons between two groups were analyzed using an unpaired Student’s *t*-test, while multiple-group comparisons were performed using one-way ANOVA followed by Dunnett’s post hoc test. A *p* value ≤ 0.05 was considered statistically significant.

## Data Availability

The original contributions presented in this study are included in the article/Supplementary Material. Further inquiries can be directed to the corresponding author.

## Supplementary Materials

The following supporting information can be downloaded at https://www.mdpi.com/article/10.3390/ijms27177870/s1. References [70,71,72,73,74,75,76,77,78,79,80,81] are cited in the supplementary materials.
