# Supplementary material for: Let-7a-5p/SHIP-1 Axis Drives SARS-CoV-2 Spike-1-Induced Microglial Pyroptosis
Source: Int J Mol Sci. 2026 Sep 3;27(17):7870. doi: 10.3390/ijms27177870 (PMC13566090; doi:10.3390/ijms27177870)
Supplement: Supplementary file 1 [file ijms-27-07870-s001.zip › ijms-4510969-supplementary.pdf]

Table S1: Genes with forward and reverse primer sequences

Primer name	Sequence	Bases	References
β-Actin: Forward	5'-TCGTCCACCGCAAATGCTTCTAG-3'	23	[1]
β-Actin: Reverse	5'-ACTGCTGTCACCTTCACCGTTCC-3'	23	
IL-1β: Forward	5′-ATGCACCTGTACGATCACTG-3′	20	[2]
IL-1β: Reverse	5' -ACAAAGGACATGGAGAACACC-3'	21	
IL-18: Forward	5′-ATGGCTGCTGAACCAGTAGAAG-3'	22	[3]
IL-18: Reverse	5′-CAGCCATACCTCTAGGCTGGC-3'	21	
IL-6: Forward	5'-AACCTGAACCTTCCAAAGATGG-3'	22	[4]
IL-6: Reverse	5'-TCTGGCTTGTTCCTCACTACT-3'	21	
TNF-α: Forward	5'-ATGGGCTACAGGCTTGTCACTC-3'	22	[5]
TNF-α: Reverse	5'-GTTTCACGTCGTCCGTCTTCTC-3'	22	
IFN-α: Forward	5'-ATTTCTGCTCTGACAACCTC- 3'	20	[6]
IFN-α: Reverse	5'-TGACAGAGACTCCCCTGATG 3'	20	
IFN-β: Forward	5'-GGTTACCTCCGAAACTGAAGA-3'	21	[7]
IFN-β: Reverse	5'-CCTTTCATATGCAGTACATTAGCC-3'	24	
TGF-β: Forward	5′-CCCAGCATCTGCAAAGCTC-3'	19	[8]
TGF-β: Reverse	5′-GTCAATGTACAGCTGCCGCA-3'	20	
SHIP1: Forward	5′-ATGACATCGCGGAGATGGTT-3′	20	[9]
SHIP1: Reverse	5′-TCATCTGAAACTTTTCTGCTG T-3′	21	
NLRP3: Forward	5′-AGCACCAGCCAGAGTCTAAC-3′	20	[10]
NLRP3: Reverse	5′-CCCCAACCACAATCTCCGAAT-3′	21	
Caspase-1: Forward	5′-GCCTGTTCCTGTGATGTGGAG-3	21	[11]
Caspase-1: Reverse	5′-TGCCCACAGACATTCATACAGTTTC-3′	25	
TLR2: Forward	5'-CCTTCACTCAGGAGCAGCAA-3'	20	[12]
TLR2: Reverse	5'-TCAGCAGGAACAGAGCACAG-3'	20	
TLR3: Forward	5'-CACCATTCCAGCCTCTTCGT-3'	20	[12]
TLR3: Reverse	5'-CAGGGTTTGCGTGTTTCCAG-3'	20	
TLR4: Forward	5'-GGTGCCTCCATTTCAGCTCT-3'	20	[13]

TLR4: Reverse	5’-ACTGCCAGGTCTGAGCAATC-3'	20	

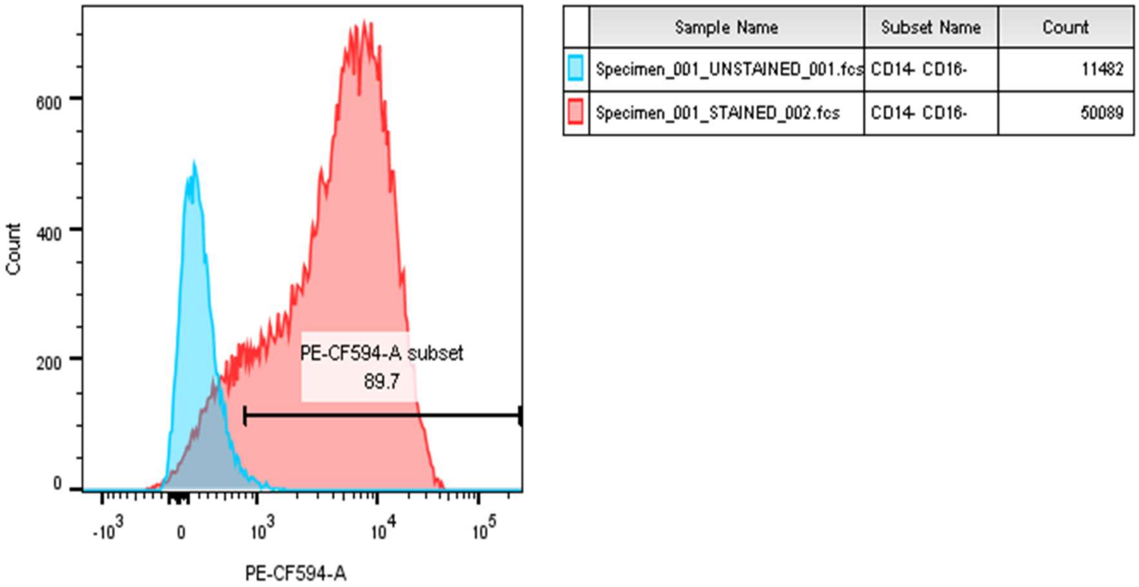

Figure S1: *Purity assessment of MDMi. Differentiated microglial cells (MDMi) were stained using PE/Dazzle™ 594 anti-human P2RY12 Antibody. The purity of microglia generated was assessed via flow cytometry using the PE-CF594-A labelled P2RY12, a microglial marker.*

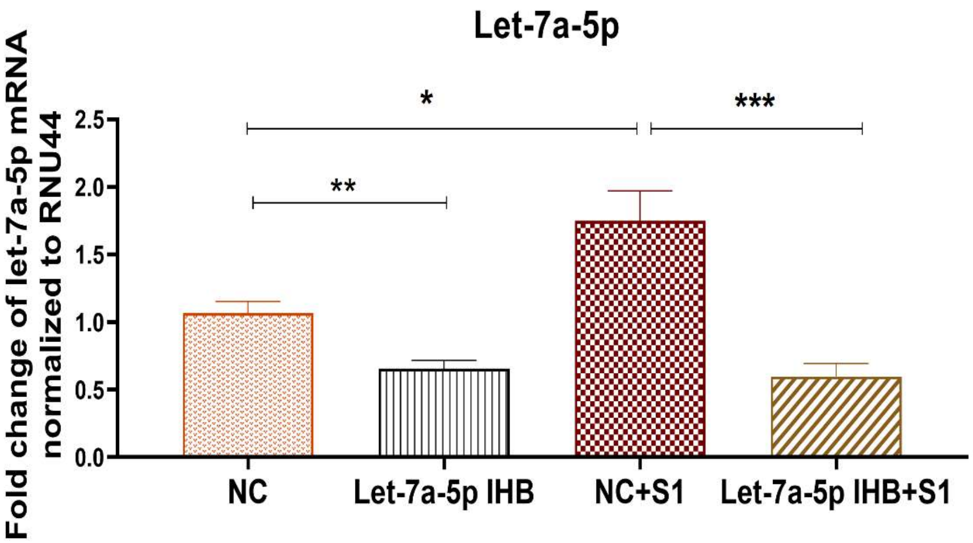

Figure S2: *Validation of let-7a-5p depletion in CHME3 cells upon let-7a-5p inhibitor treatment. CHME3 cells were transfected with 150nM of let-7a-5p inhibitor (let-7a-5p IHB) or mock scrambled miR inhibitor control (denoted as NC), followed by stimulation with SARS-CoV-2 Spike (S1) (150 ng/ml) protein (denoted as NC+S1and let-7a-5p IHB+S1). After 24h, the mRNA expression of let-7a-5p was assessed by RT-qPCR. Statistical analysis was performed using one-way ANOVA with Dunnett's multiple comparisons test; * indicates p-value<0.05, ** indicates p-value< 0.01, *** indicates p-value< 0.001, **** indicates p-value< 0.0001.*

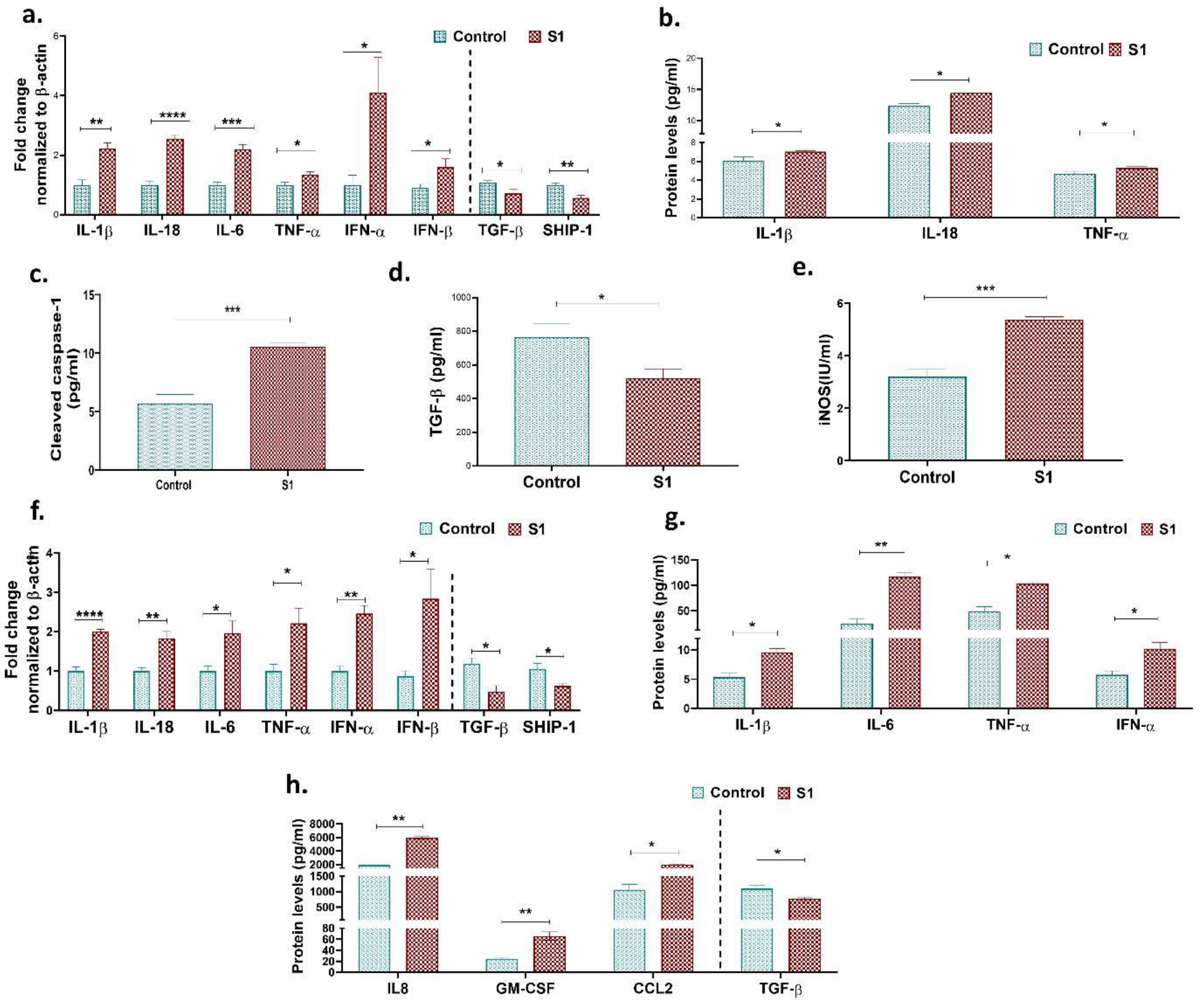

Fig S3. *SARS-CoV-2 Spike protein exacerbates proinflammatory response and oxidative stress in human microglial cells. (a, f) CHME3 cells and monocyte-derived microglial cells (MDMi) stimulated with SARS-CoV-2 S1 protein (150 ng/mL) for 24 h were used to assess the mRNA expression profile of proinflammatory cytokines IL-1β, IL-18, IL-6, TNF-α, IFN-α, IFN-*

*β, TGF-β, and SHIP-1 by RT-PCR. Data normalized to β-actin and expressed as fold change of stimulated samples (S1) over control (non-stimulated cells). (b) Protein levels of IL-1β, IL-18, TNF-α, (c) cleaved caspase-1 (d) TGF-β (e) iNOS in the culture supernatants of S1-stimulated CHME3 cells were quantified by ELISA. (g) Protein levels of IL-1β, IL-6, TNF-α, IFN-α, and (h) IL-8, GM-CSF, MCP-1/CCL2 in S1-stimulated MDMi were determined by Luminex assay and TGF-β by using ELISA. Statistical significance was calculated using Student's unpaired t-test. * indicates p value <0.05, ** indicates p value <0.01, *** indicates p value <0.001, **** indicates p value <0.0001.*

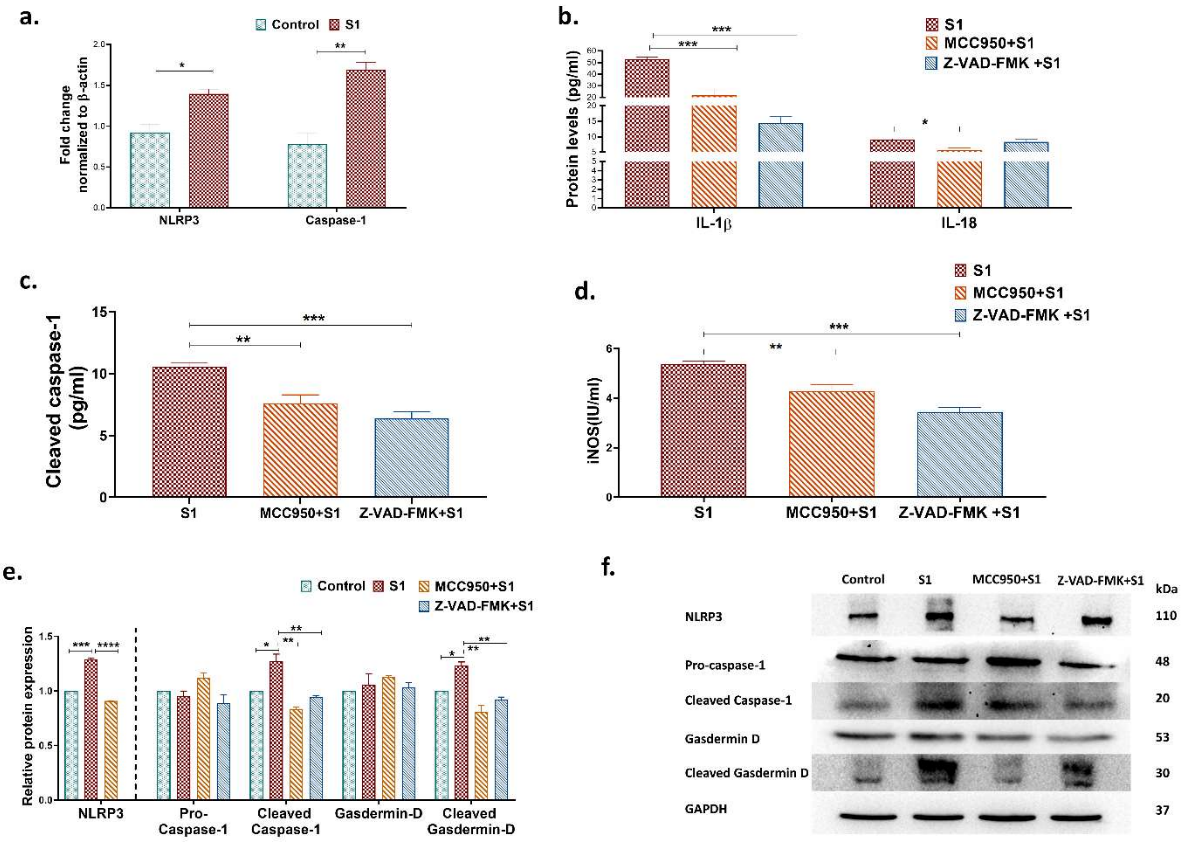

Fig.S4 *SARS-CoV-2 Spike engages inflammasome activation and pyroptosis in human microglial cells. (a) CHME3 cells stimulated with SARS-CoV-2 S1 protein (150 ng/mL) for 24 h were used to assess the mRNA expression profile of NLRP3 and cleaved caspase-1 by RT-PCR. Data are normalized to β-actin and expressed as fold change of stimulated samples (S1) over control (non-stimulated cells). CHME3 cells pre-treated with MCC950 and Z-VAD-FMK post SARS-CoV-2 S1 protein (150 ng/mL) stimulation were used to assess the (b) Protein*

*levels of IL-1β, IL-18, (c) cleaved caspase-1, and (d) iNOS were quantified by ELISA. (e, f) Western blot and densitometric analysis indicating protein expression levels of NLRP3, pro-caspase-1, cleaved caspase-1, gasdermin-D, and cleaved gasdermin-D in cell lysates of CHME3 cells treated with MCC950 and Z-VAD-FMK inhibitor post S1 stimulation are represented, respectively. Expression of GAPDH was determined in cell lysates. Fold change of S1-stimulated cells was calculated over unstimulated cells (control), while fold change of MCC950+S1 and Z-VAD-FMK+S1 treated cells was calculated over S1. Statistical significance was calculated using Student's unpaired t-test and by one-way ANOVA with Dunnett's test, * indicates p value <0.05, ** indicates p value <0.01, *** indicates p value <0.001, **** indicates p value <0.0001.*

References

1. Cai X, Zhou Y, Huang L, Zeng Q, Zhang L, Wang Q, et al. Reduced expression of Krüppel-like factor 17 is related to tumor growth and poor prognosis in lung adenocarcinoma. Biochemical and Biophysical Research Communications. 2012;418:67–73. https://doi.org/10.1016/j.bbrc.2011.12.129

2. Ali MF, Dasari H, Van Keulen VP, Carmona EM. Canonical Stimulation of the NLRP3 Inflammasome by Fungal Antigens Links Innate and Adaptive B-Lymphocyte Responses by Modulating IL-1β and IgM Production. Front Immunol. 2017;8:1504. https://doi.org/10.3389/fimmu.2017.01504

3. Kong J, Grando SA, Li YC. Regulation of IL-1 family cytokines IL-1alpha, IL-1 receptor antagonist, and IL-18 by 1,25-dihydroxyvitamin D3 in primary keratinocytes. J Immunol. 2006;176:3780–7. https://doi.org/10.4049/jimmunol.176.6.3780

4. Ecker A, Ledur PC, da Silva RS, Leal DBR, Rodrigues OED, Ardisson-Araújo D, et al. Chalcogenozidovudine Derivatives With Antitumor Activity: Comparative Toxicities in Cultured Human Mononuclear Cells. Toxicol Sci. 2017;160:30–46. https://doi.org/10.1093/toxsci/kfx152

5. Zong C, Kimura Y, Kinoshita K, Takasu S, Zhang X, Sakurai T, et al. Exposure to 1,2-Dichloropropane Upregulates the Expression of Activation-Induced Cytidine Deaminase (AID) in Human Cholangiocytes Co-Cultured With Macrophages. Toxicological Sciences. 2019;168:137–48. https://doi.org/10.1093/toxsci/kfy280

6. Chen R-F, Wang L, Cheng J-T, Yang KD. Induction of IFNα or IL-12 depends on differentiation of THP-1 cells in dengue infections without and with antibody enhancement. BMC Infectious Diseases. 2012;12:340. https://doi.org/10.1186/1471-2334-12-340

7. Vasilishina A, Kropotov A, Spivak I, Bernadotte A. Relative Human Telomere Length Quantification by Real-Time PCR. Methods Mol Biol. 2019;1896:39–44. https://doi.org/10.1007/978-1-4939-8931-7_5

8. Molnarfi N, Bjarnadóttir K, Benkhoucha M, Juillard C, Lalive PH. Activation of human B cells negatively regulates TGF-β1 production. J Neuroinflammation. 2017;14:13. https://doi.org/10.1186/s12974-017-0798-5

9. Tang H, Mao J, Ye X, Zhang F, Kerr WG, Zheng T, et al. SHIP-1, a target of miR-155, regulates endothelial cell responses in lung fibrosis. FASEB J. 2020;34:2011–23. https://doi.org/10.1096/fj.201902063R

10. Higuchi K, Sm Z, Yamashita Y, Ozaki Y, Yoshimura A. Initial periodontal treatment affects nucleotide-binding domain leucine-rich repeat-containing protein 3 inflammasome priming in peripheral blood mononuclear cells. Arch Oral Biol. 2020;110:104625. https://doi.org/10.1016/j.archoralbio.2019.104625

11. Jiang W, Lv H, Wang H, Wang D, Sun S, Jia Q, et al. Activation of the NLRP3/caspase-1 inflammasome in human dental pulp tissue and human dental pulp fibroblasts. Cell Tissue Res. 2015;361:541–55. https://doi.org/10.1007/s00441-015-2118-7

12. Li Z, Nguyen TT, Valaperti A. Human cardiac fibroblasts produce pro-inflammatory cytokines upon TLRs and RLRs stimulation. Mol Cell Biochem. 2021;476:3241–52. https://doi.org/10.1007/s11010-021-04157-7

13. Liu Z, He Y, Xu C, Li J, Zeng S, Yang X, et al. The role of PHF8 and TLR4 in osteogenic differentiation of periodontal ligament cells in inflammatory environment. Journal of Periodontology. 2021;92:1049–59. https://doi.org/10.1002/JPER.20-0285
